# 1,25-Dihydroxyvitamin D modulates L-type voltage-gated calcium channels in a subset of neurons in the developing mouse prefrontal cortex

**DOI:** 10.1038/s41398-019-0626-z

**Published:** 2019-11-11

**Authors:** Helen Gooch, Xiaoying Cui, Victor Anggono, Maciej Trzaskowski, Men Chee Tan, Darryl W. Eyles, Thomas H. J. Burne, Se Eun Jang, Manuel Mattheisen, David M. Hougaard, Bent Nørgaard Pedersen, Arieh Cohen, Preben B. Mortensen, Pankaj Sah, John J. McGrath

**Affiliations:** 1 Queensland Brain Institute, University of Queensland, St. Lucia, QLD 4072 Australia; 2 Clem Jones Centre for Ageing Dementia Research, University of Queensland, St. Lucia, QLD 4072 Australia; 3 Institute for Molecular Bioscience, The University of Queensland, St Lucia, QLD 4072 Australia; 4 Queensland Centre for Mental Health Research, The Park Centre for Mental Health, Wacol, QLD 4076 Australia; 5 Department of Biomedicine, Aarhus University, 8000 Aarhus, Denmark; 6 iPSYCH, The Lundbeck Foundation Initiative for Integrative Psychiatric Research, 8000 Aarhus, Denmark; 7 Center for Neonatal Screening, Department for Congenital Disorders, Statens Serum Institut, 2300 Copenhagen, Denmark; 8 National Centre for Register-Based Research, Aarhus University, 8000 Aarhus, Denmark; 9 Centre for Integrated Register-based Research, Aarhus University, 8000 Aarhus, Denmark; 10 Brain Research Centre and Department of Biology, Southern University of Science and Technology, Nanshan District, Shenzhen, Guangdong Province P. R. China

**Keywords:** Schizophrenia, Neuroscience

## Abstract

Schizophrenia has been associated with a range of genetic and environmental risk factors. Here we explored a link between two risk factors that converge on a shared neurobiological pathway. Recent genome-wide association studies (GWAS) have identified risk variants in genes that code for L-type voltage-gated calcium channels (L-VGCCs), while epidemiological studies have found an increased risk of schizophrenia in those with neonatal vitamin D deficiency. The active form of vitamin D (1,25(OH)_2_D) is a secosteroid that rapidly modulates L-VGCCs via non-genomic mechanisms in a range of peripheral tissues, though its non-genomic effects within the brain remain largely unexplored. Here we used calcium imaging, electrophysiology and molecular biology to determine whether 1,25(OH)_2_D non-genomically modulated L-VGCCs in the developing prefrontal cortex, a region widely implicated in schizophrenia pathophysiology. Wide-field Ca^2+^ imaging revealed that physiological concentrations of 1,25(OH)_2_D rapidly enhanced activity-dependent somatic Ca^2+^ levels in a small subset of neurons in the developing PFC, termed vitamin D-responsive neurons (VDRNs). Somatic nucleated patch recordings revealed a rapid, 1,25(OH)_2_D-evoked increase in high-voltage-activated (HVA) Ca^2+^ currents. Enhanced activity-dependent Ca^2+^ levels were mediated by L-VGCC but not associated with any changes to *Cacna1c* (L-VGCC pore-forming subunit) mRNA expression. Since L-VGCC activity is critical to healthy neurodevelopment, these data suggest that suboptimal concentrations of 1,25(OH)_2_D could alter brain maturation through modulation of L-VGCC signalling and as such may provide a parsimonious link between epidemiologic and genetic risk factors for schizophrenia.

## Introduction

Schizophrenia is a poorly understood group of mental disorders with a lifetime prevalence of 0.7%^1^. The disorder is associated with both common and rare genetic variants, as well as a range of environmental exposures. Recent genome-wide association studies have identified genetic variants of L-type voltage-gated calcium channel (L-VGCC) subunits that are associated with an increased risk for schizophrenia (*CACNA1C*, *CACNB2*, *CACNA2D1*)^2^. Evidence from whole-exome sequencing^3^ and copy number variant studies^4^ have also implicated L-VGCC-related genes as risk factors for schizophrenia. While these risk variants do not result in gross dysfunction in L-VGCCs, it is thought that they may contribute to subtle changes in the developmental expression or activity of L-VGCC subunits^5^, potentially leading to neurodevelopmental abnormalities. Indeed, modulation of L-VGCC activity during brain development has been shown to alter the extent and complexity of dendritic arborisation, as well as the developmental maturation and migration of neurons in both the hippocampus^6,7^ and cortex^8^. Moreover, deletion of *Cacna1c* during embryonic development, but not in adulthood, resulted in altered adult behavioural phenotypes relevant to schizophrenia^9^. Taken together, these findings support the hypothesis that factors that alter L-VGCC activity in the developing brain may contribute to risk of schizophrenia.

There is also a growing body of epidemiological evidence implicating developmental vitamin D deficiency as a risk factor for schizophrenia. Epidemiological studies have demonstrated that winter/spring season of birth is associated with increased risk for schizophrenia—vitamin D deficiency is most prevalent during these seasons^10^. There is also an increased risk of schizophrenia in dark-skinned migrants to Nordic and northern European countries, groups known to be at increased risk of vitamin D deficiency^11,12^. Neonatal vitamin D status has also been directly linked to an increased risk for schizophrenia. A case–control study (*n* = 848) found that neonates with vitamin D deficiency had a twofold increased risk of being diagnosed with schizophrenia in later life (Incidence Rate Ratio = 2.1; 95% confidence intervals (CIs) 1.3–3.5)^13^. This finding was recently replicated using a larger sample (*n* = 2602; Incidence Rate Ratio = 1.44; 95% CIs 1.12–1.85)^14^. The biological plausibility of vitamin D deficiency as a risk factor for schizophrenia is supported by the expression of the vitamin D receptor (VDR) and 25-hydroxyvitamin D-1α-hydroxylase (an enzyme required for 1,25-dihydroxyvitamin D (1,25(OH)_2_D) production) in the human brain^15^. Further, rodent experiments have demonstrated that transient developmental vitamin D deficiency is associated with persistent neurochemical and behavioural changes that involve biological pathways of interest to schizophrenia, including the dopamine system^16,17^.

To date, this research has been based on the assumption that the biological consequences of developmental vitamin D deficiency operated via the classical *genomic* pathways. For example, vitamin D deficiency impacts on neuronal proliferation and differentiation through transcriptional activity, which is mediated by VDR forming a heterodimer with the retinoic acid X receptor and then binding to response elements in the genome^18–22^. Long-term 1,25(OH)_2_D treatment has also been shown to confer neuroprotective effects following insult, which correlated with the downregulation of both L-VGCC mRNA expression and L-VGCC surface expression^23,24^. However, despite the documented non-genomic effects of 1,25(OH)_2_D on L-VGCC-dependent calcium influx in several tissues including bone^25^, muscle^26^ and pancreas^27,28^, its non-genomic effects within the brain remain largely unexplored. To our knowledge, only a single study has implicated 1,25(OH)_2_D with rapid changes to Ca^2+^ signalling in the brain, using liquid scintillation spectrometry to reveal 1,25(OH)_2_D-induced slice uptake of ^45^Ca^2+^ that was dependent on L-type, but not N-type, VGCC activity^29^. Based on the convergence of these genetic and environmental risk factors for schizophrenia upon L-VGCC-related mechanisms, we investigated the non-genomic effects of 1,25(OH)_2_D on neuronal L-VGCC activity in the developing brain using both functional and molecular approaches.

## Material and methods

All experimental and animal care procedures were in accordance with the Australian Code of Practice for the Care and Use of Animals for Scientific Purposes and approved by the University of Queensland Animal Ethics Committee.

### Slice preparation

Balb/c mice (P8–P12) of both sexes were deeply anaesthetised using isoflurane anaesthesia, decapitated, and the brain rapidly removed and submerged in ice-cold oxygenated artificial cerebrospinal fluid (ACSF) containing (in mM): 87 NaCl, 2.5 KCl, 25 NaHCO_3_, 25 glucose, 50 sucrose, 4 MgCl_2_, 0.5 CaCl_2_, and 1.2 NaH_2_PO_4_, pH 7.4 (95% O_2_ and 5% CO_2_). Coronal slices (300 μm) containing prefrontal cortex (PFC) were prepared for both nucleated patch recordings and Ca^2+^ imaging protocols using a Vibratome (VT1000S, Leica) and incubated at 34 °C for 30 min in ACSF, containing (in mM): 118 NaCl, 2.5 KCl, 25 NaHCO_3_, 10 glucose, 1.3 MgCl_2_, 2.5 CaCl_2_, and 1.2 NaH_2_PO_4_, pH 7.4 (95% O_2_ and 5% CO_2_). PFC neurons were visualised using an upright microscope (Olympus BX50WI, Japan) and infrared differential interference contrast optics at high magnification.

### Calcium imaging

**Fig. 1:**
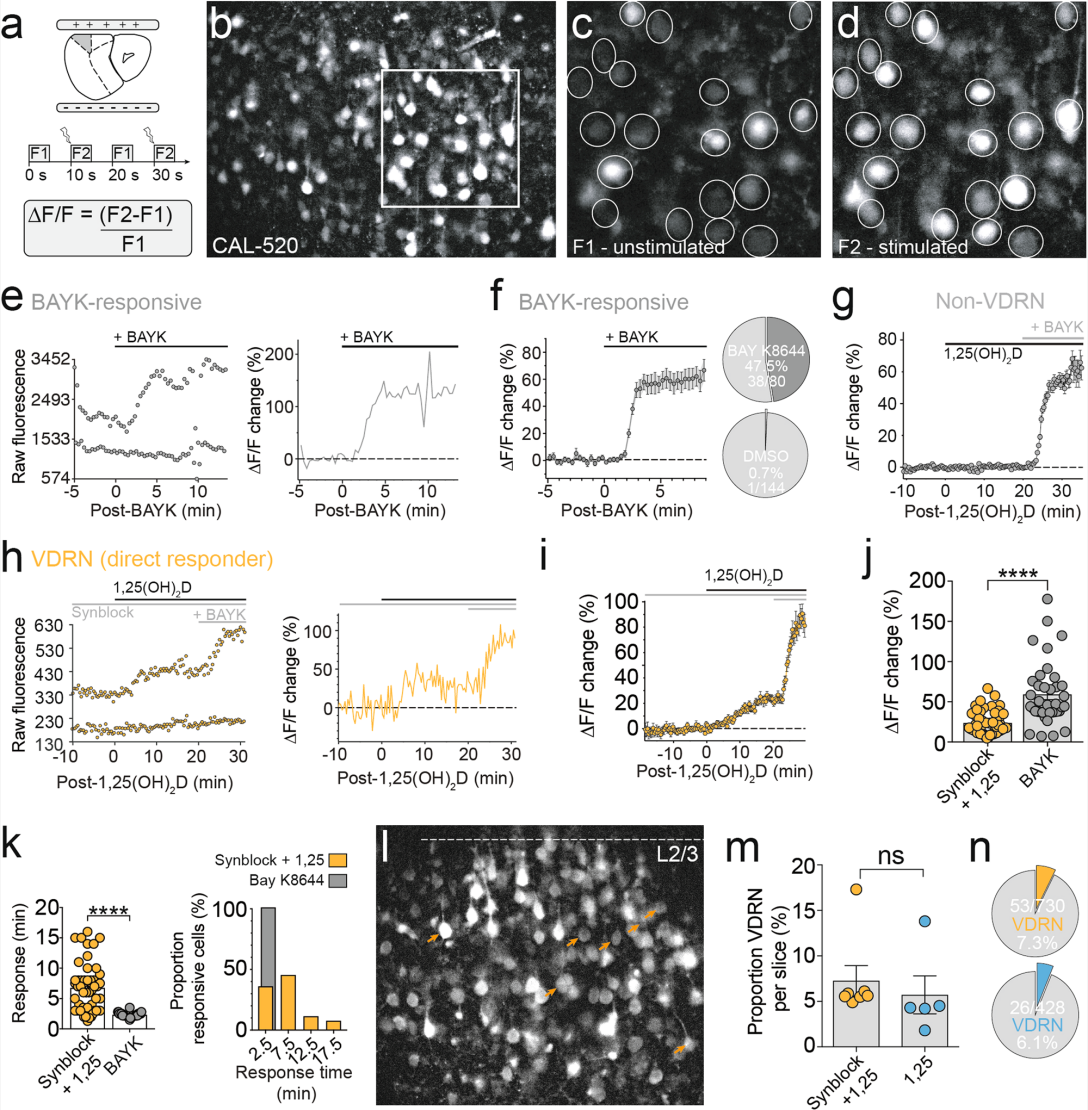
1,25(OH)_2_D enhanced activity-dependent cytosolic Ca^2+^ levels in a subset of PFC neurons. **a** Schematic of field stimulation configuration and analysis. **b** Representative PFC region bulk-loaded with Cal-520, zoomed in (white box) to show difference between unstimulated (**c**) and stimulated (**d**) fluorescence intensity in electrically responsive cells. **e** Bath application of L-VGCC agonist Bay K8644 evoked increased Ca^2+^ Δ*F*/*F* in single neurons within 3 min. **f** Bay K8644 evoked an increase in Ca^2+^ Δ*F*/*F* in almost half the imaged neurons (average Δ*F*/*F* trace, *n* = 38), while application of equimolar DMSO (10^7^-fold dilution, equivalent to dilution of 1,25(OH)_2_D stock) produced a negligible effect on Ca^2+^ Δ*F*/*F* (1/144 cells, 0.7%). **g** The majority of neurons showed no change in Ca^2+^ Δ*F*/*F* following bath application of 1,25(OH)_2_D (average Δ*F*/*F* trace, *n* = 427). **h** Raw (left) and analysed (right) Ca^2+^ imaging data from a single representative VDRN (directly responsive). **i** Average Δ*F*/*F* trace of all detected VDRN (*n* = 53). **j** 1,25(OH)_2_D induced a smaller change in the mean single cell Ca^2+^ Δ*F*/*F* compared to Bay K8644 (*p* < 0.0001). **k** Range of response times to 1,25(OH)_2_D was significantly larger compared to Bay K8644 (*p* < 0.0001). **l** Representative Cal-520 bulk-loaded slice (imaged in the presence of synaptic blockers) labelling detected VDRN with orange arrow heads. **m** Neither the percentage of VDRNs detected per imaged slice (*p* = 0.6) nor from the total pool of imaged neurons (**n**) was significantly altered by the presence (orange) or absence (blue) of synaptic blockers. Data = Mean ± SEM (unpaired two-tailed *t* test with Welch’s correction, ****p < 0.0001)

Following 25-min recovery at 34 °C, slices were loaded with Texas Red Hydrazide (TxRed; 1.8 μM; ThermoFisher Scientific; Ex/Em 582/602) for 5 min, before being transferred into standard ACSF and equilibrated to room temperature for at least 30 min. Slices were then transferred to a small volume (3 mL) incubation chamber made in-house for Cal-520 AM loading (AAT Bioquest; Ex/Em 492/514) at either room temperature or 34 °C (PhysioSuite, Kent Scientific) for 2–3 h, containing (in mM): 118 NaCl, 2.5 KCl, 25 NaHCO_3_, 10 glucose, 1.3 MgCl_2_, 2.5 CaCl_2_, and 1.2 NaH_2_PO_4_, 0.01 Cal-520, 0.03% pluronic F-127, pH 7.4 (95% O_2_ and 5% CO_2_). Slices were incubated in L-VGCC blocker nifedipine (nif; 10 μM) for >30 min prior to imaging where indicated and were imaged in alternation with bath application of 1,25(OH)_2_D alone. Following loading, slices were transferred to a submerged imaging chamber perfused with oxygenated ACSF maintained at 25 ± 1 °C (TC-324B, Warner) and secured with a platinum harp strung with parallel nylon threads. Cell depolarisation was evoked using electric field stimulation (efs) generated by two parallel platinum electrodes, separated by a distance of 5 mm, with an isolated stimulator (DS2A, Digitimer Ltd; square pulse, 1 ms, 0.05 Hz, 20–40 V). Both the imaging and stimulation systems were controlled by Metafluor (version 7.10, Molecular Devices) through a 74HC08 AND gate box (Scitech). Fluorescence imaging of the developing PFC was performed using a Polychrome V monochromator (TILL Photonics) combined with an sCMOS camera (pco.edge 5.5, PCO) and ×40 water-immersion objective, which captured a visual field of approximately 411 μm × 338 μm. Wide-field Cal-520 (50 ms) and TxRed (30 ms) fluorescent images were acquired at 0.1 Hz (2 × 2 binning), with every second of Cal-520 exposure time-locked to efs (0.05 Hz). Cal-520 and TxRed fluorescent images were collected using a multi-band dichroic and filter set (FITC/TxRed-A, Semrock; Supplementary Fig. 1a–c). TxRed images were collected to monitor for changes in *z*-depth via in-plane astrocytic processes (<3 μm), and manual depth corrections were made as required. Imaging sessions were excluded from the data set when changes in *z*-depth were observed (movement of the imaged *z*-plane towards or away from the objective lens). Instantaneous Δ*F*/*F* (0.05 Hz) was calculated for individual somatic regions of interest (ROIs) as fluorescence during field stimulation (F2) minus the immediately preceding fluorescence at rest (unstimulated, F1), divided by the fluorescence at rest (F1, Fig. 1a). Single-cell ROI fluorescence levels were extracted offline from these acquired temporal *z*-stacks using Fiji. Background subtraction (rolling ball, radius 50 pixels) and *XY* image stabilisation were applied using built-in plugins. Instantaneous Δ*F*/*F* analysis of wide-field images was automated using custom MATLAB scripts and converted to txt files that were baselined (flat or extrapolated sloping function) and quantified using the Axograph software (Axograph X, version 1.7.2). ROI data met signal-to-noise inclusion criteria if bath application of 1,25(OH)_2_D induced a percentage change in the instantaneous Δ*F*/*F* that exceeded three standard deviations of >5 min pre-1,25(OH)_2_D baseline Δ*F*/*F* amplitude. 1,25(OH)_2_D was bath applied at the physiological concentration of 0.1 nM^30^ from a 1-mM stock solution (dimethyl sulfoxide (DMSO)). Where specified, imaging sessions were conducted in the presence of synaptic blockers (*synblock*), which included 6-cyano-7-nitroquinoxaline-2-3-dione (10 μM; Tocris), D-2-amino-5-phosphonopentanoic acid (50 μM; Tocris) and picrotoxin (100 μM; Sigma-Aldrich); the L-VGCC agonist Bay K8644 (BAYK; 2.5 μM; Sigma-Aldrich); or the L-VGCC antagonist nif (10 μM; Sigma-Aldrich). The duration of an imaging session baseline was dependent on the applied pharmacology: ACSF 10–20 min, *synblock* 20 min, nif 20 min^31,32^ (Supplementary Fig. 1d). Neurons that ceased to respond to electrical stimulation following bath application of *synblock* were excluded from the total cell count imaged per slice. Only electrically responsive, TxRed-negative cells were included in vitamin D-responsive neuron (VDRN) subsets. VDRN slice *Responding %* was calculated from the total number of Cal-520-positive cells in focus per imaged area (TxRed-positive and TxRed-negative) to avoid errors stemming from inaccurate TxRed staining. As such, VDRN *Proportion Responsive (%)* statistics may underestimate the percentage of imaged neurons that were VDRNs.

### Nucleated patch recordings

High-voltage-activated (HVA) Ca^2+^ channel currents were recorded in nucleated patch configuration^33^ using barium as the charge carrier. Nucleated patches were extracted from the soma of visually identified L2/3 PFC neurons (series resistance <15 MΩ). Briefly, negative pressure (10–15 kPa; Series 475 MARK III Digital Manometer, Dwyer Instruments, IN, USA)^34^ was applied to the patch pipette in whole-cell configuration, drawing the nucleus towards the pipette tip as it was slowly withdrawn from the soma, to produce an *outside-out* patch of somatic membrane internally scaffolded by the nucleus. Following isolation of the nucleated patch, negative pipette pressure was reduced to 3–5 kPa for the remainder of the experiment, and extracellular Ca^2+^ was exchanged for Ba^2+^ (5–10 mM), containing (in mM): 115.5/110.5 NaCl, 2.5 KCl, 25 NaHCO_3_, 10 glucose, 1.3 MgCl_2_, 5/10 BaCl_2_, and 1.2 NaH_2_PO_4_, pH 7.4 (95% O_2_ and 5% CO_2_). VGCC currents were isolated pharmacologically by bath application of tetradotoxin (1 μM) and 4-aminopyridine (5 mM) to block voltage-gated sodium and voltage-gated potassium channels, respectively, which were only applied once the nucleated patch was isolated from the remnant (anucleated) neuron and situated in the external ACSF (input resistance range typically 1–5 GΩ). Recording pipettes were fabricated from borosilicate glass that was pulled and fire-polished to a tip resistance between 3 and 6 MΩ (GC150F, 1.5 mm, Harvard Apparatus, UK) when filled with caesium-based internal solution, containing (in mM): 120 CsMeSO_4_, 10 TEACl, 10 HEPES, 4 Mg_2_ATP, 0.3 Na_3_GTP, 20 phosphocreatine, and 0.3 EGTA (pH 7.3 with CsOH; osmolarity ~295–300 mOsm/kg). VGCC currents were evoked with a 50-ms depolarising pulse from −80 to 0 mV every 20 s (0.05 Hz). Current–voltage (*I*–*V*) relationships were investigated by 50-ms step depolarisations from −90 to +40 mV in 10-mV increments from a holding potential of −80 mV. Linear leak and capacitive currents were subtracted online by a P/N protocol (15 repetitions of voltage step −80 to −100 mV). 1,25(OH)_2_D was applied at the physiological concentration of 0.1 nM^30^ from a 1-mM stock solution (DMSO). Recordings were made using a MultiClamp 700B amplifier (Molecular Devices) with Sutter manipulators (MP-285). Recordings were filtered at 6 kHz and digitised at 10–50 kHz using an ITC-18 board (InstruTech, Port Washington, NY), attached to an iMac. Recordings were acquired and analysed offline using Axograph software (Axograph X, version 1.4.4).

### Statistics

Calcium imaging statistics were evaluated using unpaired two-tailed *t* test with Welch’s correction (unequal variances *t* test). All data were analysed using Prism 7 (GraphPad Software) and are reported as mean ± SEM. Additional details and experimental design are included in Supplementary Methods.

## Results

### Simultaneous measurement of single-cell VGCC activity from a population of neurons

Conventional methods for the functional investigation of VGCC activity have low spatial resolution, in that they involve single-cell recording configurations in order to provide sufficient voltage control. To locate and quantify the effects of 1,25(OH)_2_D on VGCCs in the brain, we developed a high-throughput imaging method that allowed the simultaneous measure of single-cell activity-dependent cytosolic Ca^2+^ from a population of neurons (Fig. 1a–d). This method combines wide-field Ca^2+^ fluorescence imaging with field stimulation in brain slices, thereby retaining cortical layer organisation (Fig. 1a). Cytosolic Ca^2+^ levels were visualised using the fluorescence indicator Cal-520 (50-ms exposure; 0.1 Hz; Fig. 1b), with every second exposure time-locked to field stimulation (F2; 0.05 Hz; Fig. 1c, d). This approach evoked stable activity-dependent increases in cytosolic Ca^2+^ in postnatal PFC neurons (P8–P12) throughout the imaged area, for extended imaging durations (>1 h). Bath application of dihydropyridine (DHP) L-VGCC agonist BAYK (2.5 μM) confirmed sufficient sensitivity to detect changes in L-VGCC activity (Fig. 1e), resolving changes to Δ*F*/*F* as low as 7% (59 ± 6%, *n* = 38; Fig. 1f). BAYK increased Ca^2+^ Δ*F*/*F* within 3 min of application (2.3 ± 0.05 min), which was consistent with the effect of BAYK on single-channel L-VGCC recordings^35^. Further, BAYK evoked an increased Ca^2+^ Δ*F*/*F* in 47.5% of the imaged neurons (Fig. 1f), consistent with L-VGCC protein expression in the PFC^9^. Application of equimolar DMSO (10^7^-fold dilution, equivalent to dilution of 1,25(OH)_2_D stock) evoked negligible effect on Ca^2+^ Δ*F*/*F* measured from imaged neurons (1/144 DMSO-responsive neuron, 0.7%; Fig. 1f).

### 1,25(OH)_2_D enhanced activity-dependent cytosolic Ca^2+^ levels in a subset of neurons

Using the approach described above, 1,25(OH)_2_D (0.1 nM) was bath applied to brain slices containing PFC. In order to identify neurons directly responsive to 1,25(OH)_2_D, calcium imaging was conducted in either the presence (colour-coded orange) or absence (colour-coded blue) of *synblock* (Supplementary Fig. 1d). While the majority of electrically responsive neurons were non-responsive to 1,25(OH)_2_D (Non-VDRN, Fig. 1g), a subset of PFC neurons rapidly responded to 1,25(OH)_2_D application with significantly enhanced activity-dependent Ca^2+^ levels (Fig. 1h; Supplementary Fig. 2), termed VDRNs. In the presence of *synblock* (orange), cytosolic Ca^2+^ Δ*F*/*F* levels increased by as much as 66.5% (24 ± 2%, *n* = 53; Fig. 1i, j) as early as 2 min following bath application, with the majority of VDRNs responding within 10 min (6.7 ± 0.5 min, *n* = 53; Fig. 1k). Notably, both the change in Ca^2+^ Δ*F*/*F* levels (*p* < 0.0001; Fig. 1j) and the range of response times (*p* < 0.0001; Fig. 1k) were significantly different compared to neurons responding to the direct-binding L-VGCC agonist BAYK. Application of 1,25(OH)_2_D in the absence of *synblock* did not change the 1,25(OH)_2_D-induced increase in Δ*F*/*F* (29 ± 4%, *n* = 26; *p* = 0.17) nor the onset of response in detected VDRNs (7.6 ± 1 min, *n* = 26; *p* = 0.43), suggesting 1,25(OH)_2_D did not enhance Ca^2+^ Δ*F*/*F* levels through calcium-permeable AMPA or *N*-methyl-d-aspartate receptors. Mapping directly responsive VDRNs onto their respective Ca^2+^-imaged areas revealed the scattered nature of VDRN soma localisation throughout the cortical layers of the PFC (Fig. 1l, orange arrows). There was also no significant difference (*p* = 0.6) between the proportion of cells that responded with increased Ca^2+^ Δ*F*/*F* levels in either the presence (7.3 ± 2%, *n* = 7 slices, Fig. 1m; 53/730 cells, 7.3%, Fig. 1n) or absence (slice mean = 5.7 ± 2%, *n* = 5 slices, Fig. 1m; 26/428 cells, 6.1%, Fig. 1n) of *synblock*, suggesting that VDRNs did not drive enhanced Ca^2+^ Δ*F*/*F* levels in local postsynaptic non-VDRN. In summary, wide-field calcium imaging revealed that 1,25(OH)_2_D directly enhanced activity-dependent Ca^2+^ levels in a subset of neurons scattered throughout the PFC, within minutes of application.

### 1,25(OH)_2_D enhanced HVA Ca^2+^ channel currents in a subset of PFC neurons

**Fig. 2:**
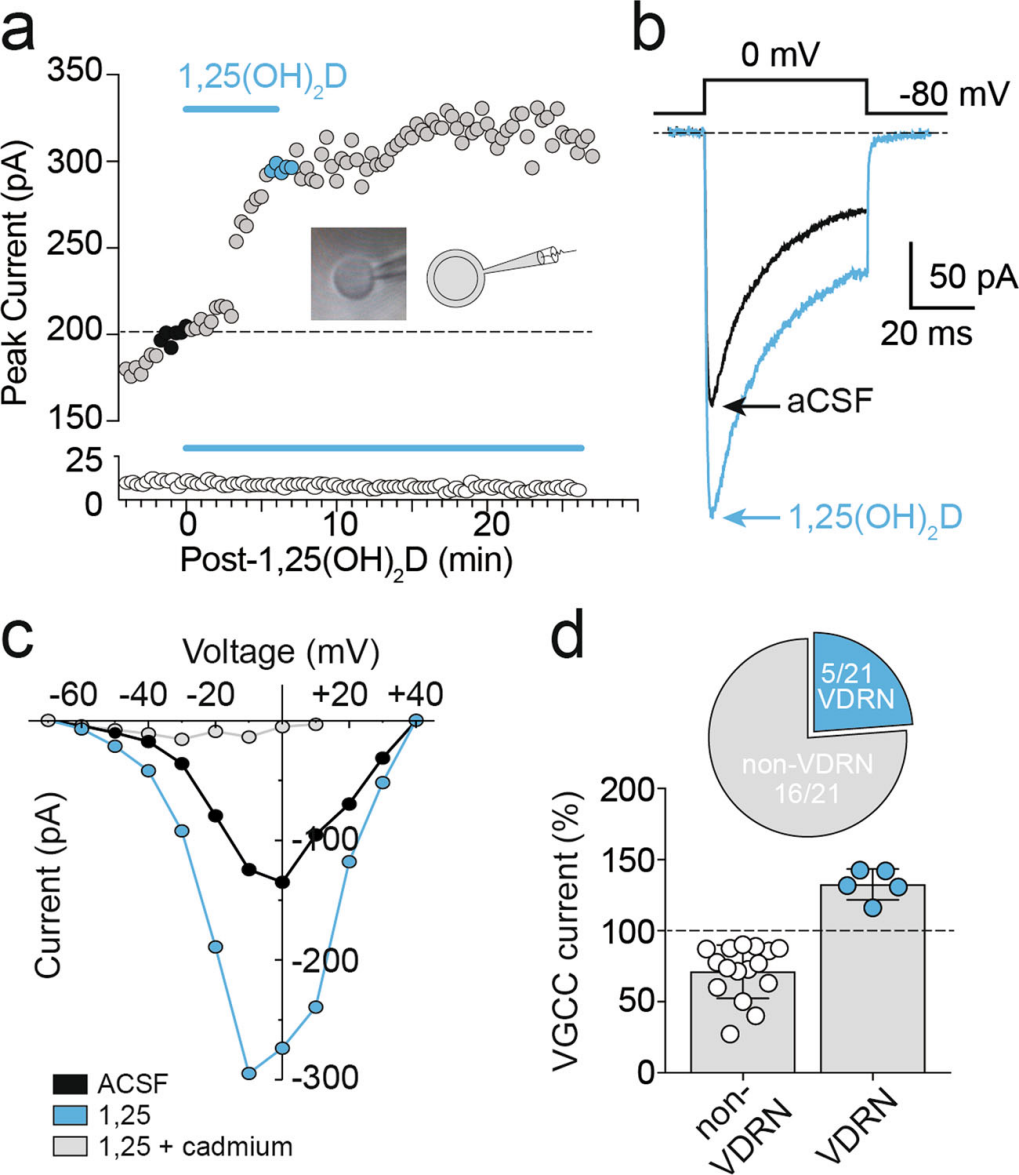
1,25(OH)_2_D enhanced high-voltage-activated (HVA) Ca^2+^ channel currents in a subset of PFC neurons. **a** Baseline VGCC current amplitudes recorded in nucleated patch configuration (averaged in **b**, black trace) rapidly increased (averaged in **b**, blue trace) during bath application of 0.1 nM 1,25(OH)_2_D in a subset of PFC neurons (above), while the majority of recorded neurons were insensitive (below). Inset, representative and schematic examples of nucleated patch configuration following extraction from whole-cell configuration. **c** VGCC *I*–*V* recorded pre- (black) and post-1,25(OH)_2_D application (blue), abolished by cadmium (1 μM, grey). **d** 1,25(OH)_2_D evoked a mean increase in the recorded HVA Ca^2+^ current amplitudes of 33 ± 5% (blue circles, *n* = 5), while the majority of cells (open circles) demonstrated peak current amplitude rundown typical of L-VGCC channels (−29 ± 5%; *n* = 16). Data = Mean ± SEM

Fluorescent Ca^2+^ indicators cannot differentiate Ca^2+^ released intracellularly from Ca^2+^ that enters a cell through channels in the plasma membrane. In order to test whether 1,25(OH)_2_D evoked changes in voltage-gated Ca^2+^ influx, we recorded HVA Ca^2+^ currents in nucleated patch configuration from layer 2/3 PFC neurons. Consistent with the above Ca^2+^ imaging findings, a subset of recorded neurons showed rapidly (3 ± 1 min onset, *n* = 5; Fig. 2a) increased voltage-gated Ca^2+^ currents (33 ± 5%, *n* = 5; Fig. 2a–d) in response to 1,25(OH)_2_D (0.1 nM; peak response by 8 ± 2 min, *n* = 5). Following 1,25(OH)_2_D application, currents were entirely blocked by the broad-spectrum Ca^2+^ channel blocker cadmium (1 μM; Fig. 2c) to confirm that only HVA Ca^2+^ currents were evoked. Typical of L-VGCC currents, and despite the inclusion of high phosphocreatine in the recording pipette, the majority of nucleated patch recordings showed rundown in HVA Ca^2+^ current amplitudes^36,37^ (Fig. 2a, d, white circles).

### Enhanced activity-dependent Ca^2+^ levels mediated by L-type VGCCs

**Fig. 3:**
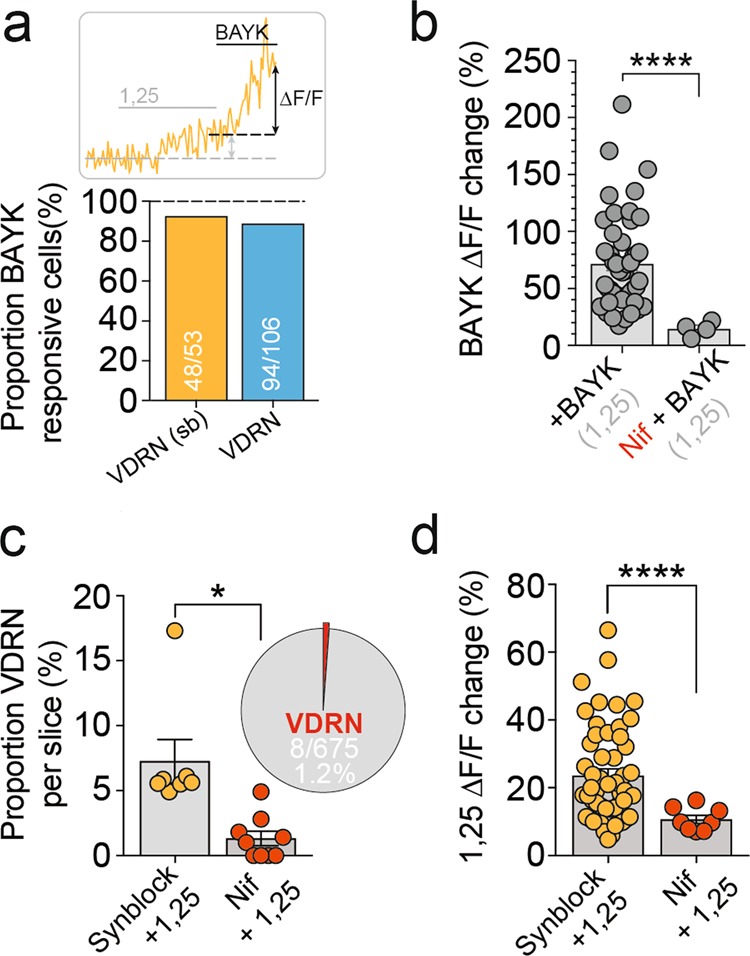
Enhanced activity-dependent Ca^2+^ levels mediated by L-type VGCCs. **a** The majority of VDRNs showed an increased Ca^2+^ Δ*F*/*F* in response to L-VGCC agonist Bay K8644, suggesting functional L-VGCC expression. **b** In the presence of 1,25(OH)_2_D, Bay K8644 increased VDRN Ca^2+^ Δ*F*/*F* in a manner comparable to Bay K8644 alone (Fig. 1i) and was attenuated by preincubation in L-VGCC antagonist nifedipine (*p* < 0.0001). Pre-incubation of imaged slices in nifedipine significantly reduced both the proportion of detected VDRN (**c** *p* = 0.01) and magnitude of detected Ca^2+^ Δ*F*/*F* changes in response to 1,25(OH)_2_D (**d** *p* < 0.0001). Data = Mean ± SEM (Unpaired two-tailed *t* test with Welch’s correction, *p < 0.05, ****p < 0.0001)

Since 1,25(OH)_2_D enhanced HVA Ca^2+^ influx in a subset of PFC neurons (Fig. 2), and 1,25(OH)_2_D is known to modulate L-type VGCC activity in peripheral tissues, we considered whether 1,25(OH)_2_D enhanced activity-dependent Ca^2+^ levels by rapidly modulating L-VGCC activity. We first assessed whether VDRN expressed functional L-VGCCs by bath applying L-VGCC-specific agonist BAYK (2.5 μM^38^) following 1,25(OH)_2_D application. While BAYK evoked a rapid Ca^2+^ Δ*F*/*F* increase in approximately half the total imaged neurons (Fig. 1h), the vast majority of VDRNs (48/53, 91%; Fig. 3a) showed a significant increase in Ca^2+^ Δ*F*/*F* following BAYK application. Conversely, approximately 12% of BAYK-responsive neurons were VDRNs (53/438 neurons; 12.7 ± 3%, *n* = 7 slices). In the presence of 1,25(OH)_2_D, BAYK evoked a similar increase in Ca^2+^ Δ*F*/*F* (72 ± 6%, *n* = 48, *p* = 0.15; Fig. 3b) compared to application of BAYK alone (59 ± 6%, *n* = 38; Fig. 1f), suggesting no facilitative or depressive interaction between the two molecules.

We next tested whether 1,25(OH)_2_D enhanced activity-dependent Ca^2+^ levels in VDRN through modulation of L-VGCC activity. Slices were preincubated with L-VGCC-specific blocker nif (10 μM; >30 min), and baseline calcium imaging was conducted in the presence of nif for a further 20 min (electrically stimulated 60 times at 0.05 Hz; Supplementary Fig. 1d) to maximise the state and depolarisation dependency of DHP antagonism efficacy^31,32^. Significantly, preincubation in nif almost entirely abolished 1,25(OH)_2_D-induced changes to Ca^2+^ Δ*F*/*F*, attenuating both the proportion of 1,25(OH)_2_D-responsive neurons (8/675, 1.2%, *n* = 9 slices; *p* = 0.01; Fig. 3c) and the magnitude of detected Ca^2+^ Δ*F*/*F* changes (10.7 ± 1%, *n* = 8 cells, *n* = 9 slices; *p* < 0.0001; Fig. 3d). Interestingly, preincubation in nif attenuated BAYK-induced increases in VDRN Ca^2+^ Δ*F*/*F* to a similar extent (14 ± 3%, *n* = 4 cells, *n* = 9 slices; *p* < 0.0001; Fig. 3b), suggesting that residual 1,25(OH)_2_D-induced changes to Ca^2+^ Δ*F*/*F* (Fig. 3d) resulted from the incomplete antagonism of nif^32,38,39^. These findings suggest that 1,25(OH)_2_D enhanced activity-dependent Ca^2+^ levels in a distinct subset of developing PFC neurons through rapid modulation of L-VGCC activity. Further, this effect was not associated with changes to *Cacna1c* mRNA levels (Supplementary Fig. 3), suggesting a non-genomic mechanism.

## Discussion

**Fig. 4:**
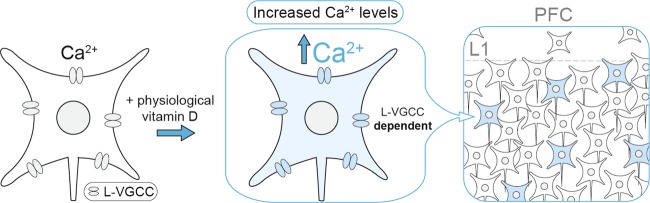
A non-genomic role for 1,25(OH)_2_D in the developing brain. VDRNs responded rapidly to 1,25(OH)_2_D treatment with increased Ca^2+^ influx mediated by L-VGCCs. Through their modulation of L-VGCC, optimal levels of 1,25(OH)_2_D may contribute to the healthy development of VDRNs within the maturing cortical circuit, such as through neurite extension, neuronal excitability, and gene expression

Vitamin D deficiency, a prevalent exposure in many parts of the world^40^, reduces the expected (basal) concentration of 1,25(OH)_2_D^41^. While developmental vitamin D deficiency is associated with a range of altered brain outcomes^19^, the assumption to date has been that these properties were mediated via classical genomic pathways (i.e., involving the nuclear receptor VDR). Here we provide evidence that 1,25(OH)_2_D also affects brain function via rapid, non-genomic mechanisms. Physiological levels of 1,25(OH)_2_D rapidly enhanced Ca^2+^ influx through L-VGCCs in a small subset of neurons in the PFC, leading to elevated intracellular calcium levels during neural activity (Fig. 4). We propose that suboptimal concentrations of this secosteroid during critical periods of brain development may result in altered L-VGCC function in VDRNs. Since L-VGCC activity is critical to healthy neurodevelopment^7^, vitamin D deficiency may disrupt the excitability and maturation of VDRNs, with possible consequences for local circuit integration, function, and information processing. This non-genomic mechanism may represent a link between two independent genetic and epidemiologic risk factors for schizophrenia.

### 1,25(OH)_2_D non-genomically modulated L-VGCC activity in a subset of PFC neurons

We show with single-cell resolution that 1,25(OH)_2_D rapidly enhanced activity-dependent Ca^2+^ Δ*F*/*F* through L-VGCCs in a subset of developing PFC neurons. Changes to L-VGCC-mediated Ca^2+^ influx can be evoked through (1) modulation of single-channel conductance or gating, such as through DHP agonists and antagonists^38,42^ and/or (2) regulation of the number of channels present in the plasma membrane via protein trafficking. Interestingly, the kinetics of this effect were different to those evoked by L-VGCC agonist BAYK, where 1,25(OH)_2_D induced a smaller mean change to Ca^2+^ Δ*F*/*F*, with a slower mean response time, and was not observed in all neurons that expressed L-VGCCs. This difference likely reflects an alternative mechanism of action compared to BAYK, which binds directly to the pore-forming α1 subunit of L-VGCCs to enhance channel gating. Further, this finding indicates that only a subset of L-VGCC-expressing neurons contained the machinery required for initiation of 1,25(OH)_2_D-evoked effects, which may suggest the involvement of specific auxiliary L-VGCC subunits^43,44^ or second messenger signalling pathways. Indeed, a previous study showed that non-genomic effects of 1,25(OH)_2_D in neurons are dependent on Ca^2+^/calmodulin-dependent protein kinase II (CaMKII) and phosphoinositide 3-kinase (PI3K)^29^, where both CaMKII and P13K have previously been shown to promote trafficking of L-VGCC neurons through the β_2_ subunit^45,46^. The propensity of these second messengers to facilitate L-VGCC activity through the β_2_ subunit is noteworthy here, since variants of the β_2_ gene (CACNB2) have also been associated with an increased risk for schizophrenia^2,3^. In order to determine whether 1,25(OH)_2_D enhances L-type VGCC Ca^2+^ influx in VDRNs by increasing Ca_V_1.2 surface expression levels, future studies will require live imaging of Ca_V_1.2 channel trafficking with single-cell resolution. Future studies should also determine: (1) the identity and role of VDRNs in the PFC, (2) whether altered L-VGCC activity during developmental vitamin D deficiency affects VDRN properties and circuit function, and (3) the distribution of VDRNs outside the PFC and at other stages of development.

### L-VGCCs, brain development and schizophrenia

L-VGCCs are highly expressed in the developing brain and are widely known to play a critical role in neuronal development^7^, neuronal excitability, synaptic plasticity, homoeostatic plasticity and the transduction of neuronal activity into gene expression^47^. L-VGCC activity plays a critical role in mediating cortical neurite extension and radial migration^8^, and both ionic and conformational signalling in L-VGCC are required to drive neuronal gene expression^48^. Indeed, disruption to L-VGCC function in hippocampal parvalbumin interneurons during development significantly reduced cell number and dendritic arbor complexity^7^. L-VGCCs are also critical in the regulation of basal and burst firing activity in dopaminergic neurons of the ventral tegmental area^49^, which are considered responsible for the control of basal dopamine levels in downstream areas^50^, and are also the target of significant PFC projections^51^. Notably, both parvalbumin interneurons and the dopamine system are implicated in current hypotheses in schizophrenia research^52^. Critically, disruption to L-VGCC activity has also been implicated in behavioural phenotypes of interest to psychiatry. A recent transgenic animal study suggested that deletion of *Cacna1c* in forebrain glutamatergic neurons during embryonic development, but not adulthood, was associated with hyperactivity, impaired hippocampal synaptic plasticity, cognitive impairment and reduced sociability^9^. These findings support the hypothesis that factors influencing L-VGCC activity in the developing brain may contribute to risk of schizophrenia.

In addition to schizophrenia^3^, variants in L-VGCC-related genes such as *CACNA1C* and *CACNB2* have also been linked to a range of other mental disorders^53^. In particular, variants in L-VGCC-related genes have been linked to the risk of autism-related phenotypes^54^. Recent studies based on the Generation R birth cohort have found that gestational vitamin D deficiency is also associated with an increased risk of both autism-related traits and autism-spectrum disorder^55,56^. These findings are in keeping with the expectation that genetic and environmental risk factors are often shared across a range of psychiatric phenotypes.

Genetic and epidemiological studies have provided important insights into the aetiology of schizophrenia; however, it is rare that discoveries from these fields converge on a shared neurobiological pathway. Indeed, studies that can combine genetics, epidemiology and basic neuroscience are considered better able to triangulate on causal mechanisms^57^. This approach led us to investigate the links between two established risk factors for schizophrenia: risk variants in L-VGCC genes and developmental vitamin D deficiency. Using functional approaches, we have demonstrated that vitamin D rapidly enhanced L-VGCC activity in a subset of PFC neurons during brain development. We propose that optimal concentrations of 1,25(OH)_2_D contribute to the normal (expected) development and maturation of VDRNs through the non-genomic modulation of L-VGCC activity. Conversely, we propose that suboptimal concentrations of 1,25(OH)_2_D may disrupt developmentally critical L-VGCC-dependent processes within the immature cortical circuit. In other words, vitamin D deficiency may produce a transient *channelopathy-like state*, in that the activity of L-VGCCs is altered during critical periods of neurodevelopment. However, unlike genetic diseases that affect L-VGCC activity, vitamin D deficiency is preventable with supplementation. Unravelling the underlying mechanism, as well as the identity and role these neurons play within the circuit, may provide further insight into the aetiology and pathophysiology of schizophrenia.

## Supplementary information

Supplement
