# Supplementary material for: 1,25-Dihydroxyvitamin D modulates L-type voltage-gated calcium channels in a subset of neurons in the developing mouse prefrontal cortex
Source: Transl Psychiatry. 2019 Nov 11;9:281. doi: 10.1038/s41398-019-0626-z (PMC6848150; doi:10.1038/s41398-019-0626-z)
Supplement: Supplementary file 1 — Supplement [file 41398_2019_626_MOESM1_ESM.docx]

**1,25 dihydroxyvitamin D modulates L-type voltage-gated calcium channels in a subset of neurons in the developing mouse prefrontal cortex**

**Supplementary Material**

*Contents:*

1. Supplementary Methods
2. Supplementary Figures
3. Additional references

**Supplementary Methods:**

*Primary neuronal cultures*

Cortical neurons were prepared from cortices of embryonic day 18 rat pups as described previously ^1-4^. Briefly, dissociated cells, which consist of mainly neurons and a small percentage of astrocytes, were plated onto poly-L-lysine-coated dishes in Neurobasal medium (Thermo Fisher Scientific, MA USA) supplemented with 2% B27 (Invitrogen Life Technology, MA, USA), 2 mM Glutamax, 50 U/ml penicillin, 50 μg/ml streptomycin, and 1% fetal bovine serum (FBS) (Hycone Laboratories, Utah, USA). Neurons were seeded at 15 x 10^4^ cells per well in a 12-well plate. Fresh media replaced the initial plating media 6 h post seeding to eliminate apoptotic cells and debris. Neurons were maintained in Neurobasal medium and fed twice a week. At *in vitro* day 5 (DIV5), 5’-fluoro-2’-deoxyuridine (FDU), a potent inhibitor of thymidylate synthetase, was added to the growth medium to inhibit the proliferation of astrocytes. Neurons at DIV14 were treated with 1,25(OH)_2_D (20 nM, Merck Millipore, VIC Australia) for 5, 10 and 30 minutes prior to RNA extraction.

*RNA extraction, cDNA synthesis and real-time quantitative PCR*

Total RNA was extracted using the RNeasy Mini Kit (Qiagen) according to the manufacturer’s protocol (Life Technologies). cDNAs were then generated using QuantiTect reverse-transcription kit (Qiagen, MD, USA). To quantify changes in mRNA expression, SYBR green-based qPCR reaction (Qiagen) were performed on the CFX384 Real-Time qPCR Detection System (Bio-Rad). Gene expression was calculated using the standard comparative Ct method by normalizing to the housekeeping gene β-actin. The sequences of primers for Ca_V_1.2 and β-actin were previously published ^5, 6^: Ca_V_1.2 primer 1 forward 5’- CGAAGGTACATCCCCAAGAA-3’; reverse 5’-CGATTTTGAAGAGGCAGCTC-3’; β-actin primer forward 5’-GTGACGTTGACATCCGTAAAGA-3’; reverse 5’-GCCGGACTCATCGTACTCC-3’. Ca_V_1.2 primer 2 forward 5’- CAGCTCATGCCAACATGAAT-3’; reverse 5’- TGCTTCTTGGGTTTCCCATA-3’.^7^ GAPDH primer forward 5’-TGCCCCCATGTTTGTGATG-3’; reverse 5’-TGTGGTCATGAGCCCTTCC-3’.^4^ The PCR conditions were as follows: a denaturation step at 95°C for 10 min, followed by amplification for 40 cycles (95°C for 10s, and 60°C for 1 min).

*Experimental design and statistical analysis*

Our objective was to identify, quantify and characterise the effects of vitamin D on neuronal calcium homeostasis. For nucleated patch recordings and wide-field calcium imaging experiments, the response to 1,25(OH)_2_D was tested once per slice. For calcium imaging, vitamin D responsive neurons were included in analysis if 1,25(OH)_2_D induced a percentage change in the instantaneous ΔF/F that exceeded three standard deviations of >5 mins pre-1,25(OH)_2_D baseline ΔF/F amplitude. The proportion of vitamin D responsive neurons was calculated per slice (from the total number of Cal-520 positive cells), as well as per condition (pooled independent slice experiments under the same pharmacological condition). Three pharmacological conditions were imaged for comparison: (1) vitamin D alone (n = 428 cells, n = 5 slices, n = 5 animals, n = 4 litters), (2) vitamin D in the presence of synaptic blockers (n = 730 cells, n = 7 slices, n = 5 animals, n = 3 litters), (3) vitamin D in the presence of nifedipine (n = 675 cells, n = 9 slices, n = 8 animals, n = 4 litters). The number of imaged or recorded neurons included in each experiment is listed in Results, and the responses of individual neurons are included in graphical representations. All statistical values were evaluated using unpaired two-tailed *t*-test with Welch’s correction, and exact *p* values are provided in the corresponding results and figure legends. All data were analysed using Prism 7 (GraphPad Software) and are reported as mean ± SEM.

**Supplementary Figure Legends:**

***Supplementary Figure 1****. Wide-field calcium imaging.*

(**a-c**) Spectral separation of Cal-520 and TxRed fluorescent images was achieved with a multi-band dichroic and filter set. (**d)** Schematics of bath applied pharmacology used during calcium imaging assays.

***Supplementary Figure 2****.* *1,25(OH)_2_D treatment enhanced activity-dependent cytosolic Ca^2+^ levels in a subset of primary cortical neurons.*

(**a**) Schematic of field stimulation configuration and analysis. (**b**) Representative cortical culture bulk-loaded with Cal-520. (**c**) Raw (*left*) and analysed (*right*) Ca^2+^ fluorescence signals demonstrate that electric field stimulation evoked calcium influx during cell depolarisation that was blocked by tetrodotoxin (TTX, 1 μM). (**d**) Raw (*left*) and analysed (*right*) Ca^2+^ imaging data from a single representative VDRN in primary cortical culture. (**e**) 1,25(OH)_2_D (20 ± 7%, *n* = 14) induced a smaller change in the mean single cell Ca^2+^ ΔF/F compared to Bay K8644 (42 ± 5%, *n* = 44; *p* < 0.0001). (**f**) Range of response times to 1,25(OH)_2_D (7 ± 1 min, *n* = 14) was significantly larger compared to Bay K8644 (3 ± 0.1 min, *n* = 44; *p* < 0.0001). (**g**) Representative coverslip of Cal-520 bulk-loaded primary cortical neurons, labelling detected VDRN with blue arrow heads. Data = Mean ± SEM (Unpaired two-tailed Mann-Whitney test, *****p* < 0.0001).

***Supplementary Figure 3****.* *1,25(OH)_2_D treatment did not alter the levels of Cacna1c mRNA.*

Rat cortical neurons at DIV 14 were treated with 1,25(OH)_2_D (20 nM) for 5, 10 and 30 minutes, and *Cacna1c* mRNA was assessed using real time PCR using two sets of *Cacna1c* primers normalised against two house-keeping genes. (**a**) *Cacna1c* primer 1 versus β-actin. (**b**) *Cacna1c* primer 2 versus β-actin. (**c**) *Cacna1c* primer 1 versus GAPDH. (**d**) *Cacna1c* primer 2 versus GAPDH. 1,25(OH)_2_D had no effect on *Cacna1c* mRNA levels at any of the tested time points.

**Supplementary Figure 1.** *Wide-field calcium imaging.*


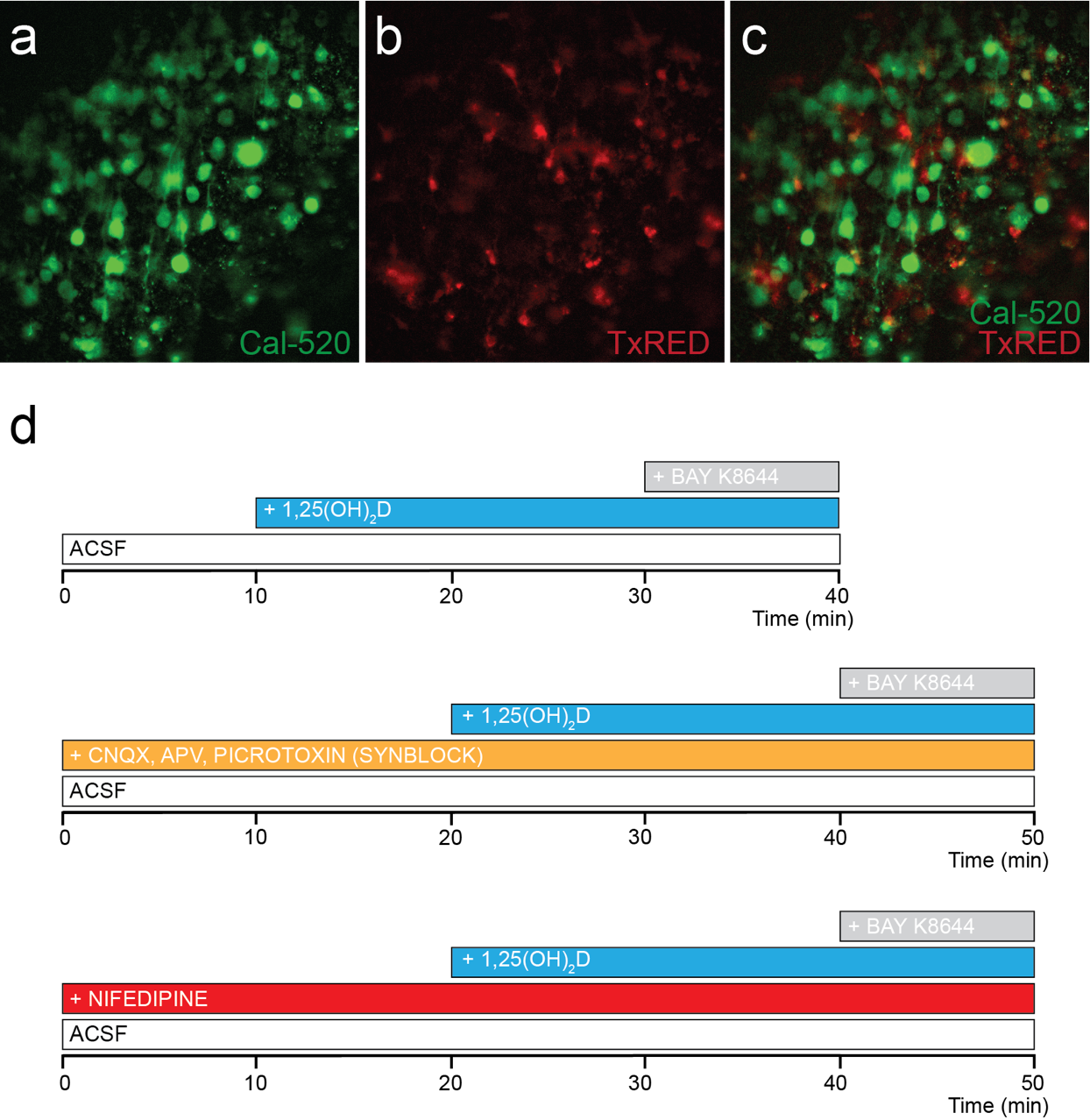


**Supplementary Figure 2.** *1,25(OH)_2_D treatment enhanced activity-dependent cytosolic Ca^2+^ levels in a subset of primary cortical neurons*

**
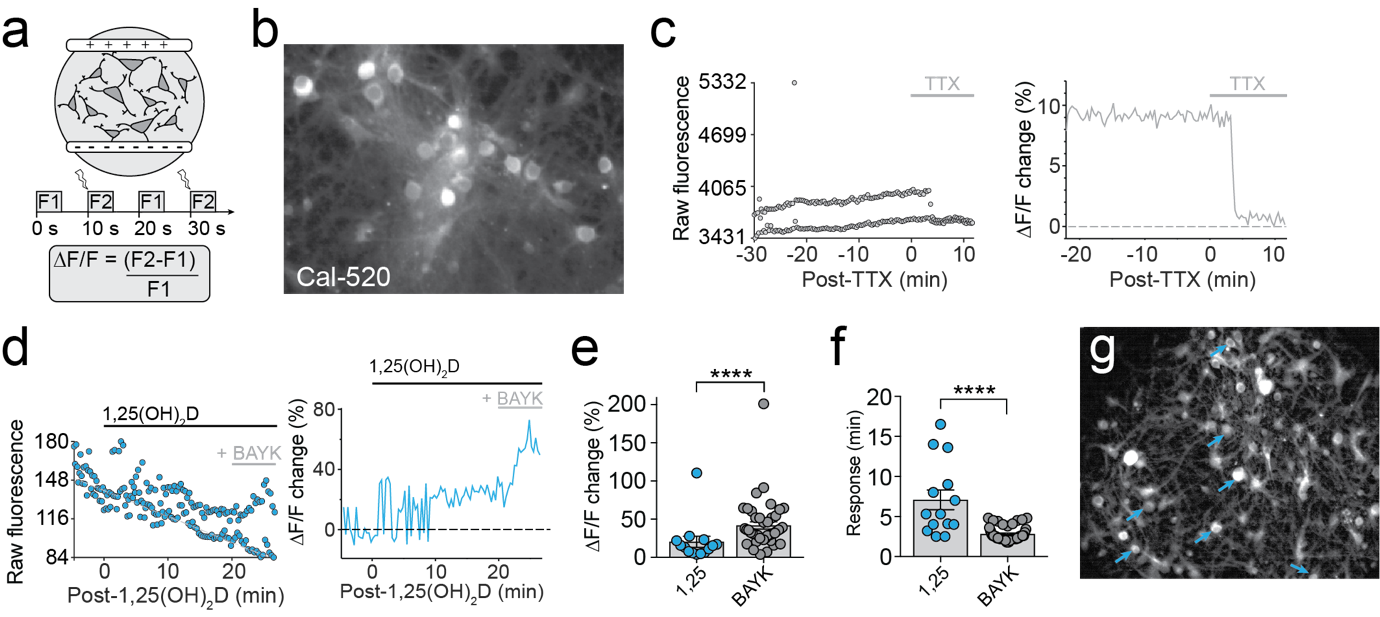
**

**Supplementary Figure 3.** *1,25(OH)_2_D treatment did not alter the levels of Cacna1c mRNA.*

**
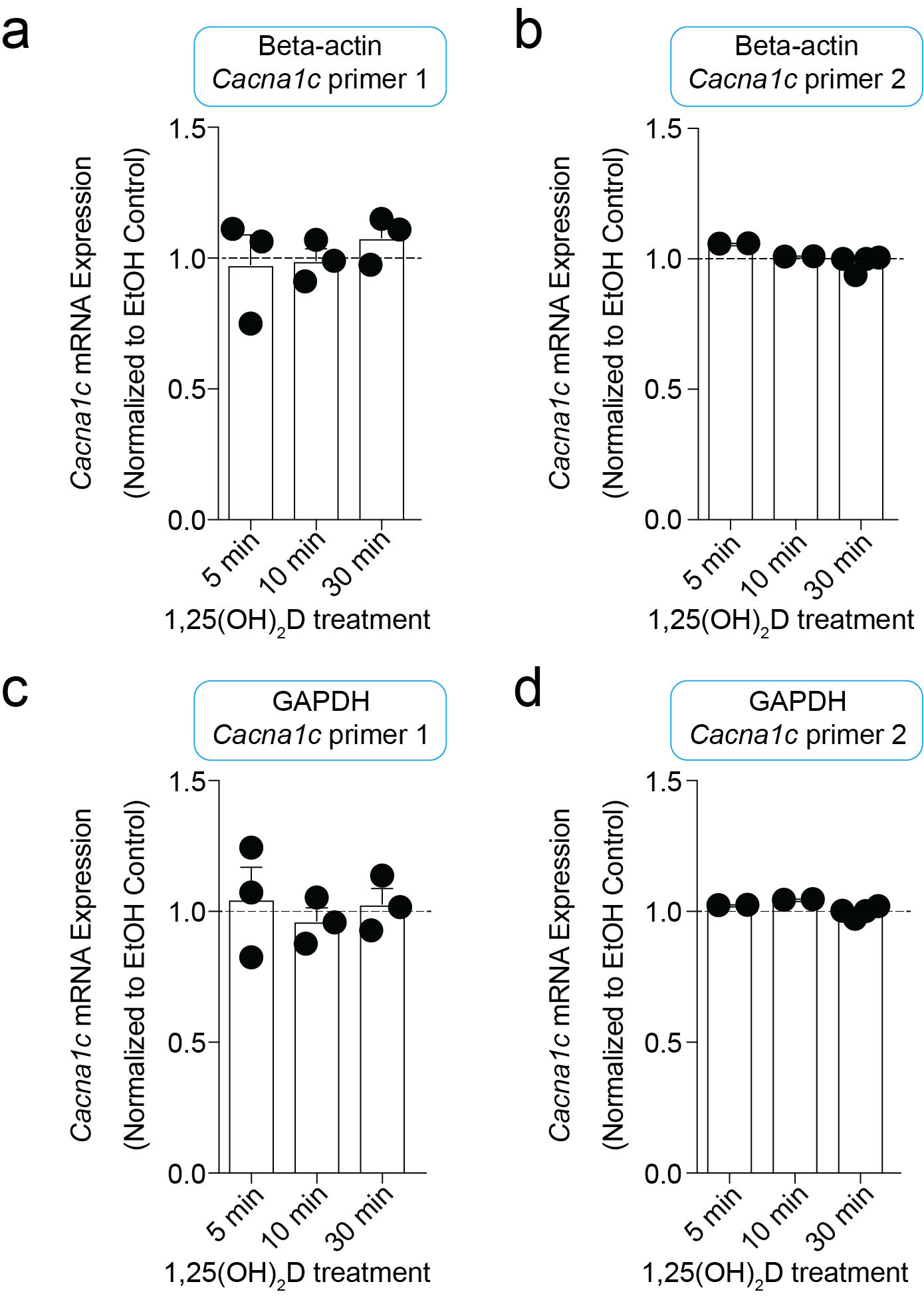
**

**References**

1. Widagdo J, Zhao QY, Kempen MJ, Tan MC, Ratnu VS, Wei W *et al.* Experience-Dependent Accumulation of N6-Methyladenosine in the Prefrontal Cortex Is Associated with Memory Processes in Mice. *The Journal of neuroscience : the official journal of the Society for Neuroscience* 2016; **36**(25)**:** 6771-6777.

2. Widagdo J, Chai YJ, Ridder MC, Chau YQ, Johnson RC, Sah P *et al.* Activity-Dependent Ubiquitination of GluA1 and GluA2 Regulates AMPA Receptor Intracellular Sorting and Degradation. *Cell Rep* 2015.

3. Guntupalli S, Jang SE, Zhu T, Huganir RL, Widagdo J, Anggono V. GluA1 subunit ubiquitination mediates amyloid-beta-induced loss of surface alpha-amino-3-hydroxy-5-methyl-4-isoxazolepropionic acid (AMPA) receptors. *J Biol Chem* 2017; **292**(20)**:** 8186-8194.

4. Tan MC, Widagdo J, Chau YQ, Zhu T, Wong JJ, Cheung A *et al.* The Activity-Induced Long Non-Coding RNA Meg3 Modulates AMPA Receptor Surface Expression in Primary Cortical Neurons. *Front Cell Neurosci* 2017; **11:** 124.

5. Cooper G, Lasser-Katz E, Simchovitz A, Sharon R, Soreq H, Surmeier DJ *et al.* Functional segregation of voltage-activated calcium channels in motoneurons of the dorsal motor nucleus of the vagus. *J Neurophysiol* 2015; **114**(3)**:** 1513-1520.

6. Priya A, Johar K, Nair B, Wong-Riley MT. Specificity protein 4 (Sp4) regulates the transcription of AMPA receptor subunit GluA2 (Gria2). *Biochimica et biophysica acta* 2014; **1843**(6)**:** 1196-1206.

7. Xu JH, Long L, Tang YC, Hu HT, Tang FR. Ca(v)1.2, Ca(v)1.3, and Ca(v)2.1 in the mouse hippocampus during and after pilocarpine-induced status epilepticus. *Hippocampus* 2007; **17**(3)**:** 235-251.
